# Supplementary material for: An Explainable AI Exploration of the Machine Learning Classification of Neoplastic Intracerebral Hemorrhage from Non-Contrast CT
Source: Cancers (Basel). 2025 Jul 29;17(15):2502. doi: 10.3390/cancers17152502 (PMC12346390; doi:10.3390/cancers17152502)
Supplement: Supplementary file 1 [file cancers-17-02502-s001.zip › cancers-3750338-supplementary.pdf]

Supplementary Material

Data preparation

As described in our previous publication [S1], for ICH segmentation, the region of interest (ROI) histogram was sampled within the range of 20 to 80 Hounsfield units (HU), excluding voxels likely associated with cerebrospinal fluid or calcification. The ROI histogram for PHE was sampled within the range of 0 to 30 HU, excluding voxels likely corresponding to leucariosis. The delineation of ROIs was performed using the MITK Workbench 2016.11.0 Software. The Dice Similarity Coefficient (DSC) was used to assess the quality of the automatic deep-learning segmentations and compare it to the intra-rater human reference. The DSC for the ICH was high with a mean of 0.87±0.02 for the deep-learning model and 0.83±0.05 for the human reference. Mean DSC values dropped slightly for the PHE with a mean of 0.67±0.02 and 0.67±0.07 for the model and the human reference, respectively.

Summary of previous work

This study builds directly on our previously published work[S2], in which we developed a deep learning model for the classification of neoplastic versus non-neoplastic intracerebral hemorrhage (ICH) based on admission non-contrast CT. The current study does not involve retraining or modifying this model. Instead, we apply explainable AI (XAI) techniques to analyze the trained model's decision-making process.
To provide context and assist readers, we summarize here the most relevant details of the original classification model. For comprehensive information, please refer to the full publication.

Model Architecture and Training

A ResNet-34 convolutional neural network was used for classification. The dataset was split into 90% training and 10% testing. Hyperparameter tuning was conducted to optimize performance for the classification task. The final model was trained for 182 epochs using a batch size of 64, a learning rate of 0.000296, and the Adam optimizer. The loss function was Negative Log-Likelihood, and image augmentation was performed using the batchgenerators library.

Model Evaluation

Model performance was evaluated using the area under the curve (AUC) and detailed case-by-case analysis of false positives and false negatives. A human benchmark was established using three radiologists with varying levels of expertise in ICH imaging. The model achieved classification performance comparable to that of an expert with extensive experience in stroke imaging and research in neoplastic ICH.

Limitations Relevant to the Current Study

One key limitation relevant to this XAI-based analysis is that the classification model was trained on data from a single center. In the previous work, we observed that multi-center training significantly improved performance for the segmentation model; a similar effect is likely for classification. Multi-center training to enhance generalizability and robustness remains an important direction for future work. Additionally, future research may explore alternative model architectures (e.g., transformer-based classifiers) and assess whether explanation methods produce similar attribution patterns across architectures.

Explanation Methods

Saliency

Saliency is defined as the gradients of the output with regard to the input. A gradient is a vector which indicates the direction and magnitude of the highest increase. Imagine standing on a steep

road; the gradient would point in the direction of the steepest next step you could take from this position. In the context of machine learning, gradients are calculated by measuring how sensitive the model's predictions are to changes in each input feature. If increasing the value of a feature (eg a pixel) tends to increase the prediction value, the gradient for this feature would be positive. Conversely, if increasing the feature tends to decrease the prediction value, the gradient for the feature would be negative. High gradients can, therefore, be thought of as having a high impact on the outcome, which means they are probably important for the model's decision process. In this method, the gradients are calculated using a first order Tayler network expansion.
A heat map generated with the saliency method typically shows sharp, often noisy regions highlighting areas that have the highest gradient values. This method tends to focus on the pixels with the strongest direct influence on the model's output but may lack clarity due to noisy gradients.

InputXGradient

Similar to the saliency method, InputXGradient takes the gradients of the model in respect to the input as well. Additionally, these are multiplied with the input itself. In a linear model, this would correspond to multiplying the coefficients of each feature with the value of the feature to obtain the overall contribution. The same logic can be applied to images, where the features are the pixel values, and the coefficients are the gradients of the output with respect to the input.
The heat map produced by InputXGradient is generally smoother compared to Saliency, as it incorporates both input and gradients. This helps to reveal more relevant features while filtering out irrelevant noise, creating a more focused map.

SmoothGrad

SmoothGrad is a gradient-based attribution method as well. In order to reduce the uncertainty and random variations that can occur in gradient-based visualizations, SmoothGrad generates multiple noisy versions of the input by adding random noise to it. Then, the gradients for these perturbations are calculated and averaged. By taking the average of the gradients, the random noise tends to cancel out, resulting in a more stable and smoother heatmap.
SmoothGrad's heat map is noticeably smoother compared to Saliency and InputXGradient. By averaging over noisy inputs, it creates a more refined visualization that highlights important regions without sharp edges, offering a clearer view of relevant features.

Gradient Shap

Gradient Shap bases its attribution method on the idea of Shapley values from cooperative game theory. In cooperative game theory, Shapley values are used to determine the fair distribution of payoffs among players in a coalition game. Each player's contribution to the overall outcome is measured, and Shapley values allocate the total payoff in a way that rewards individual contributions fairly.
In a similar way, it is adapted to machine learning in order to distribute the contribution of each input feature to the overall prediction. They are obtained by calculating the differences in the prediction across all possible feature subsets and averaging them.
For images, this is extremely computationally expensive, therefore the values are approximated by randomly sampling from a distribution between the baseline and the actual input rather than computing every possible permutation.
This technique generates heat maps that tend to have more nuanced and stable feature attributions. Unlike Saliency or InputXGradient, it produces smoother and more balanced maps, accounting for interactions between input features and model output.

GradCam

GradCam, short for Gradient-weighted Class Activation Mapping, works by calculating the gradients of the model's output with respect to the convolutional feature maps of a selected convolutional layer. In our case, the first convolutional block was selected, which is closest to the input image. The gradients are then combined with the feature maps using element-wise multiplication to yield the attribution map.

GradCam's heat maps focus on high-level regions in the image, highlighting areas of the image that activate certain filters in the network's last convolutional layer. It often shows broader areas of importance (like the general shape or object) rather than fine details.

Guided GradCam

In Guided GradCam, the process begins with calculating the gradients of the model's output with respect to the convolutional feature maps of a selected layer, similar to GradCam. These gradients represent the importance of each feature map in making the final prediction. However, instead of directly combining these gradients with the feature maps, Guided GradCam incorporates guided backpropagation to refine the attribution process. During the guided backpropagation step, only positive gradients are considered, ensuring that the gradients reflect the contribution of relevant features while suppressing noise and irrelevant information.

The Guided GradCam heat map refines GradCam by introducing more precise edge details, making the heat map more detailed and localized. It combines the advantages of GradCam's spatial importance with the finer details of Guided Backpropagation.

GradCam++

GradCam++ is an extension of the GradCam technique that addresses the issue of localization accuracy by leveraging positive and negative gradients during the calculation of importance weights. The main difference between GradCam and GradCam++ lies in the way they calculate the importance weights or gradients for generating the heatmap.

Instead of averaging the gradients as in GradCam, GradCam++ utilizes a guided backpropagation process that considers the positive and negative gradients separately. This approach helps in preserving more precise localization information and generating sharper heatmaps.

GradCam++ improves upon GradCam by better capturing weak and multiple object regions. Its heat map is more refined, showing finer distinctions in feature importance and offering clearer visual insights, especially in complex images with overlapping objects.

Supplementary References

[S1] Jawed Nawabi, *et al.* "Cross-institutional automated multilabel segmentation for acute intracerebral hemorrhage, intraventricular hemorrhage, and perihematomal edema on CT." Radiology Advances, Volume 2, Issue 2, March 2025, doi: 10.1093/radadv/umaf012

[S2] Nawabi, Jawed, *et al.* "End-to-end machine learning based discrimination of neoplastic and non-neoplastic intracerebral hemorrhage on computed tomography." Informatics in Medicine Unlocked 54 (2025): 101633, doi.org:10.1016/j.imu.2025.101633
